# Comparison of micro-radiofrequency therapy and tolterodine for the treatment of newly diagnosed overactive bladder: A retrospective cohort study

**DOI:** 10.3389/fnins.2023.1120843

**Published:** 2023-03-20

**Authors:** Min Tang, Jin Liu, Chesong Zhao, Chengming Wang, Qian Zhang, Mulong Du, Xiaoxin Meng, Pu Li

**Affiliations:** ^1^Department of Urology, The First Affiliated Hospital of Nanjing Medical University, Nanjing, Jiangsu, China; ^2^Clinical Medicine Research Institution, The First Affiliated Hospital of Nanjing Medical University, Nanjing, Jiangsu, China; ^3^Department of Biostatistics, Center for Global Health, School of Public Health, Nanjing Medical University, Nanjing, Jiangsu, China

**Keywords:** micro radiofrequency therapy, tolterodine, overactive bladder, frequent urination, urgent urination

## Abstract

**Purpose:**

This study aimed to retrospectively compare the efficacy and safety of micro-radiofrequency (RF) therapy through the urethra vs. oral tolterodine tartrate in the treatment of newly diagnosed overactive bladder (OAB).

**Materials and methods:**

In this study, 46 patients who were newly diagnosed with moderate-to-severe OAB were included; 23 of them underwent the micro-RF treatment procedure, and the other 23 patients took tolterodine. Bladder diaries were recorded 3 days before treatment and during the follow-up period on 1, 3, and 7 weeks after micro-RF therapy or oral tolterodine. Micturition parameters including daily voiding times, daily urge urinary incontinence (UI) episodes, daily urgency episodes, mean volume per micturition, post-void residual volume (PVR), maximum urine flow rate (Qmax), overactive bladder symptom score (OABSS), and quality of life (QoL) score were analyzed.

**Results:**

All 46 patients underwent either micro-RF or oral tolterodine treatment, as well as a complete follow-up. The incidence of adverse events in the micro-RF group was 8.7% (2/23), and that in the tolterodine group was 43.5% (10/23). The following two adverse events happened in the micro-RF group: an injury to the urethra during catheterization in a man and a urinary tract infection in a woman, both of which were relieved or disappeared after day 3. The adverse effects in the tolterodine group were mainly dry mouth (4/23), dysuria (5/23), and constipation (8/23), but none of the patients withdrew from the drug therapy. Compared to pre-therapy, all parameters of both groups, including daily voiding times, daily urgency episodes, mean volume per micturition, OABSS, and QoL score, demonstrated significant improvements during follow-up in 7 weeks after therapy, except for daily UI episodes in the tolterodine group, while the above parameters showed bigger improvements in the micro-RF group than in the tolterodine group. Besides, the general treatment efficacy of micro-RF was 73.9% (17/23), which was significantly better than tolterodine (10/23, 43.5%), and the difference was 30.4% [95% CI: 3.4–57.5%, *p* = 0.036].

**Conclusion:**

In this retrospective study, we found that micro-RF therapy is safe and more effective than oral tolterodine for newly diagnosed moderate-to-severe OAB in a short-term follow-up. Stronger evidence would be provided through a well-designed, prospective, randomized controlled trial.

## 1. Introduction

Overactive bladder (OAB) is defined as a sudden, compelling desire to void urine, with or without urge incontinence (UI), accompanied by frequent urination and nocturia (Abrams et al., 2002). It was reported that the overall prevalence of OAB in community-dwelling adults was between 12 and 17% and increased with age in both men and women (Markland et al., 2010; Irwin et al., 2011). The OAB symptoms affect not only patients' quality of life (QoL) but also an increased incidence rate of anxiety and depression (Milsom et al., 2012).

The first-line treatment for OAB is behavior therapy, followed by pharmacological management with anti-muscarinic or β3-adrenoreceptor agonist drugs as second-line treatment. More invasive therapies such as bladder injection of botulinum toxin type A and sacral neuromodulation are considered third-line treatments (Lightner et al., 2019). However, behavior therapy as an intervention combining pelvic floor muscle training and urge suppression strategies to improve bladder control could not completely cure the symptoms and thus had poor patient compliance (Chapple et al., 2008). Drug therapy inevitably causes various adverse reactions in different systems, including gastrointestinal, cardiac, neurological, urogynecological, and nasopharyngeal systems (Truzzi et al., 2016; White and Iglesia, 2016), and is hence limited in practice. Sacral neuromodulation and other surgical treatments are often not acceptable due to invasiveness and high cost. These limitations lead to a lack of effective options for refractory patients with OAB to improve their quality of life.

Micro-radiofrequency (RF) therapy is an innovative transurethral procedure that uses multipolar μRfthera technology to deliver a max power of 2.5 W to the bladder wall and thus reduce the sensitivity of the submucosal nerve endings of the bladder at a temperature below 45°C (Razzaghi et al., 2021). As a relatively non-invasive approach, micro-RF therapy has already shown good safety and efficacy for patients with refractory OAB who are not responding well to medication (Xu et al., 2022). However, the therapeutic effect of micro-RF therapy in patients with newly diagnosed OAB is still not clear. Therefore, we conducted this study to retrospectively compare the safety and efficacy of micro-RF therapy and tolterodine for patients with newly diagnosed moderate-to-severe OAB.

## 2. Materials and methods

### 2.1. Patient selection and pre-therapy assessment

Between November 2021 and October 2022, 46 patients with newly diagnosed moderate-to-severe OAB were enrolled in this retrospective cohort study. The diagnosis of OAB was confirmed by the clinical history, including the primary complaints, surgical history, chronic coexisting disease, and the overactive bladder symptom score (OABSS): mild OAB: OABSS ≤ 5; moderate OAB: 6 ≤ OABSS ≤ 11; severe OAB: OABSS ≥ 12 (Homma et al., 2006; Salaffi et al., 2022). Urinary ultrasound was performed to exclude patients with secondary OAB, free urine flow rate and post-void residual volume (PVR) were examined to rule out bladder outlet obstruction (BOO), and urinalysis was done to eliminate hematuria and urinary tract infection. Of note, 23 patients with moderate-to-severe OAB accepted micro-RF therapy (two times separated by 7 days), and the other 23 patients chose to take tolterodine (2 mg po bid for 7 weeks). Bladder diaries for 3 consecutive days were recorded before the beginning of the therapy, and the data were checked by urologists. All procedures performed in this study were in accordance with the Declaration of Helsinki (as revised in 2013), and this study was approved by the Ethics Committee of the First Affiliated Hospital of Nanjing Medical University (No. 2022-SR-569). Written informed consent for the publication of patient information was obtained. Table 1 shows a detailed list of inclusion and exclusion criteria.

**Table 1:** The inclusion and exclusion criteria.

Inclusion criteria	Age ≥ 18 years
	Patients newly diagnosed with moderate to severe OAB
	Normal upper urinary tract function, bladder capacity >100 ml
	Patients with persistent OAB who have not been on medication or other therapies
Exclusion criteria	Pregnant or lactating women
	Patients with secondary OAB symptoms such as urinary tract obstruction
	Patients with uncontrolled urinary system infection within 1 week
	Patients with renal insufficiency and serum creatinine >1.5 times of normal value
	Patients with any implanted neurostimulator, cardiac pacemaker, or implantable defibrillator
	Presence of concomitant diseases seriously affecting health, such as malignant tumor
	Patients who have received botulinum toxin treatment in the past 12 months
	Patients allergic to latex materials

### 2.2. Micro-RF therapy procedure

**Figure 1:**
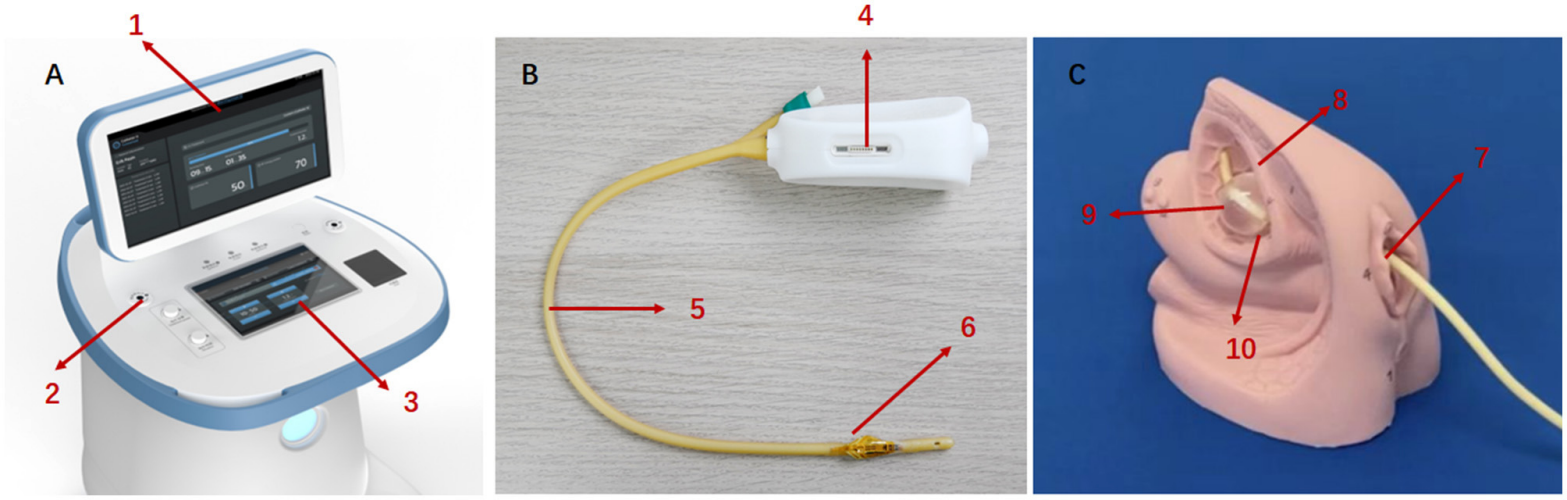
The key components of micro-RF therapy product and working scheme. **(A)** Therapeutic apparatus. 1: Display. 2: RF output connect. 3: Keyboard. **(B)** The single-use modified catheter. 4: Port of RF input. 5: Catheter. 6: Therapeutic electrodes **(C)** working scheme: through therapeutic electrodes integrated with the surface of the urinary catheter, micro-radiofrequency energy is delivered to the nerve fibers under the bladder wall, blunting part of the nerve endings temporarily. 7: Urethral orifice. 8: Bladder. 9: Catheter balloon. 10: Therapeutic electrodes.

During the treatment procedure, the patient was in a supine position, and the catheterization was completed by the same urologist (MT). The RF cable connected the catheter *via* the connector to the RF output connector of the therapeutic apparatus. The urologist ran the instrument for 20 min of treatment (5 min for each power: 0.5, 1.0, 1.5, and 2.0 W) with the temperature below 45°C, along with observing and asking the patient whether there was discomfort. After the treatment, the urologist turned off the machine, disconnected the electrodes, and removed the modified catheter immediately. Every patient had micro-RF therapy two times, at week 0 and week 1. The product was validated by the Zhejiang Medical Device Testing Institute and China Food and Drug Administration (Report Nos. Z20183035-D and Z20182736) prior to the study. The structural composition of the product and working scheme is shown in Figure 1.

### 2.3. Post-therapy follow-up

The micro-RF treatment was administered at week 0 and week 1, and tolterodine was taken for 7 consecutive weeks. Follow-up was at 1, 3, and 7 weeks, and the primary evaluation efficacy endpoint was at the end of week 7. Bladder diaries (including daily voiding times, daily urgency episodes, daily UI episodes, and mean volume per micturition), OABSS, QoL score, and urinalysis were evaluated for each follow-up, and adverse events were recorded.

### 2.4. Efficacy evaluation

The primary efficacy evaluation index was the success rate.


$$\begin{array}{l} Success\;rate=\frac{the\;number\;of\;successful\;cases}{total\;number\;of\;cases}\times 100\% \end{array}$$

Patients completed bladder diaries 3 days before each visit or treatment at week 1, week 3, and week 7. The primary efficacy evaluation was comparing the improvement from baseline at the 7-week follow-up. Success was defined as meeting any of the following criteria, including:

- ≥50% decrease or normalization (<8 times/day) from baseline in the average number of 24-h micturitions;
- ≥50% decrease or normalization (none) from baseline in the average number of 24-h urge incontinence;
- ≥50% decrease or normalization (none) from baseline in the average number of 24-h urgency.

The secondary efficacy evaluation index was the change from baseline in mean volume per micturition per 24 h, OABSS, and QoL.

### 2.5. Statistical analysis

Normally distributed continuous variables were presented as means with standard deviation and analyzed using Student's *t*-test; non-normally distributed variables were presented as medians with lower and upper quartiles and analyzed using the Wilcoxon test to compare the two groups or the Wilcoxon signed-rank test to compare pre- and post-therapy. Categorical parameters were presented as frequency (percentage) and compared using χ^2^ test or exact test when appropriate. A *P*-value of <0.05 was considered statistically significant. All the analysis was performed using SPSS 24.0.

## 3. Results

### 3.1. Baseline characteristics

A total of 46 cases were enrolled with informed consent in this retrospective study. There were no significant differences in the baseline characteristics (in terms of age, gender, height, weight, blood pressure, and duration of disease) between the two groups (Table 2). Overall, 84.8% of patients were women. Patient compliance was 100% in both groups.

**Table 2:** Baseline characteristics of the patients.

	**Micro-RF group (*n* = 23)**	**Tolterodine group (*n* = 23)**	***T*** **/χ^2^/*Z***	***P***
Age (year)	43 ± 16.17	39.17 ± 12	0.91	0.37
Sex, F/M	19/4	20/3	–	1.00
Height (m)	1.63 ± 0.07	1.66 ± 0.06	1.73	0.09
Weight (kg)	60.21 ± 7.45	58.43 ± 7.45	−0.81	0.42
Systolic blood pressure	126 ± 11.93	129.69 ± 11.51	1.07	0.29
Diastolic blood pressure	75.35 ± 9.05	79.65 ± 10.14	1.52	0.14
Daily voiding times	17 (15, 20)	16 (14, 21)	0.55	0.55
Daily urgency episodes	3 (1, 3)	2 (2, 3)	0.30	0.77
Daily UI episodes	0 (0, 1)	0 (0, 1)	−0.10	0.92
Duration of disease (year)	3.5 (2, 8)	3 (1.5, 4.5)	−0.78	0.43
**OAB severity**				
Moderate	17	19	0.51	0.48
Severe	6	4		
PVR (ml)	16 (0, 24)	15 (0, 20)	−0.83	0.41
Qmax (ml/s)	21.07 ± 2.68	22.65 ± 3.34	1.78	0.08

Data are presented as mean ± SD or median (P_25_, P_75_).

–Represent exact test.

Qmax, maximum urine flow rate; PVR, post-void residual volume.

### 3.2. Therapeutic efficacy

**Figure 2:**
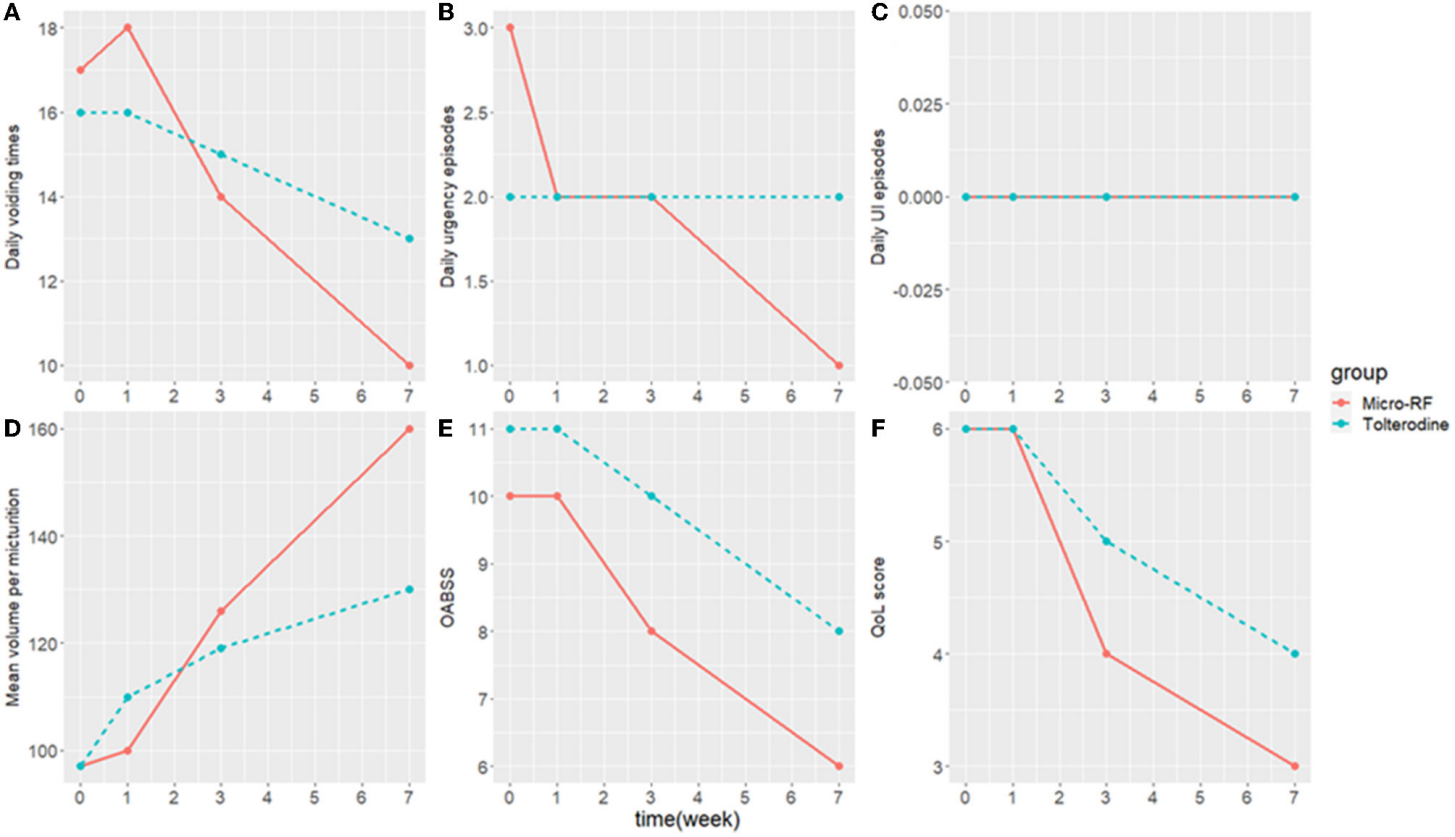
The median of the clinical outcome from baseline through 1-, 3-, and 7-week follow-up visits. **(A)** Daily voiding times. **(B)** Daily urgency episodes. **(C)** Daily UI episodes. **(D)** Mean volume per micturition. **(E)** OABSS. **(F)** QoL score. OABSS, overactive bladder symptom score; QoL score, quality of life score; UI, urinary incontinence.

The primary efficacy was evaluated with the success rate. At the end of week 7, the success rate of the micro-RF therapy group was 73.9% and that of the tolterodine group was 43.5%; the difference was 30.4% [95% CI: 3.4–57.5%, *p* = 0.036]. In the micro-RF group, one patient successfully met all three criteria, 11 patients met two indicators, and five patients successfully met one criterion. Comparatively, none of the patients met all three success criteria in the tolterodine group, but three patients met two indicators, and seven patients met one criterion. It showed that the micro-RF group had a better therapeutic effect than the tolterodine group. The urination parameters, including daily voiding times, daily urgency episodes, daily UI episodes, OAB severity, PVR, and Qmax, were similar between the two groups prior to treatment, and the differences were not statistically significant (Table 2). As Table 3 shows, both groups demonstrated significant improvement in daily voiding times, daily urgency episodes, mean volume per micturition, OABSS, and QoL score. However, there was no improvement in the daily UI episodes after treatment in the tolterodine group at the end of week 7. In detail, compared with baseline, the median decrease in the daily voiding time after therapy was significant both in the micro-RF group (−8 times, *p* < 0.01) and the tolterodine group (−5 times, *p* < 0.01), and the decrease range was significantly larger in the micro-RF group compared with the tolterodine group (−8 times vs. −5 times, *p* < 0.01). Besides, the median decrease in daily urgency episodes after therapy was significant in the micro-RF group (−2 times, *p* < 0.05), as well as in the tolterodine group (−1 time, *p* < 0.05), and the decrease range was also obviously larger in the micro-RF group than the tolterodine group (−2 times vs. −1 times, *p* = 0.02). However, daily UI episodes after therapy did not show a clinically significant change in the tolterodine group (0, *p* > 0.05), but showed an obvious drop in the micro-RF group (0, *p* < 0.05). Furthermore, the median volume per micturition after therapy showed significant increases both in the micro-RF group (65 ml, *p* < 0.01) and the tolterodine group (27 ml, *p* < 0.01). In addition, the increased range was significantly larger in the micro-RF group compared with the tolterodine group (65 vs. 27 ml, *p* < 0.01). The median of OABSS in the micro-RF group decreased significantly by 4 points (*p* < 0.05) compared to 2 points (*p* < 0.05) in the tolterodine group, and the micro-RF group had a significantly larger decrease compared with the tolterodine group (−4 vs. −2 points, *p* < 0.01). The QoL scores in the micro-RF group also improved significantly, with the median decreasing by 3 points (*p* < 0.01). In the tolterodine group, the median of the QoL decreased by 1 point (*p* < 0.05). The difference between the two groups is significant (−3 vs. −1 points, *p* = 0.04). In general, the abovementioned parameters showed bigger improvements in the micro-RF group than in the tolterodine group. Besides, as Figure 2 shows, urination parameters including daily voiding times, daily urgency episodes, mean volume per micturition, OABSS, and QoL score in the micro-RF group presented a more obvious trend of improving than the tolterodine group from baseline through 1-, 3-, and 7-week follow-up, except for daily UI episodes.

**Table 3:** Comparisons of the urination parameters before and after treatment in the two groups.

	**Daily voiding times**		**Daily urgency episodes**		**Daily UI episodes**		**Mean volume per micturition**		**OABSS**		**QoL score**	
	**Micro-RF**	**Tolterodine**	**Micro-RF**	**Tolterodine**	**Micro-RF**	**Tolterodine**	**Micro-RF**	**Tolterodine**	**Micro-RF**	**Tolterodine**	**Micro-RF**	**Tolterodine**
Pre-therapy (W0)	17 (15, 20)	16 (14, 21)	3 (1, 3)	2 (2, 3)	0 (0, 1)	0 (0, 1)	97 (75, 115)	97 (84, 121)	10 (9, 12)	11 (9, 11)	6 (5, 6)	6 (6, 6)
Post-therapy (W7)	10 (9, 11)	13 (11, 15)	1 (0, 1)	2 (1, 2)	0 (0, 0)	0 (0, 0)	160 (145, 182)	130 (110, 150)	6 (5, 7)	8 (7, 10)	3 (2, 3)	4 (3, 5)
Post-pre	−8 (−10, −5)^*^	−5 (−7, −2)^*^	−2 (−2, −1)^*^	−1 (−2, 0)^*^	0 (−1, 0)^*^	0 (0, 0)	65 (51, 87)	27 (16, 54)	−4 (−5, −2)^*^	−2 (−4, 0)^*^	−3 (−4, −2)^*^	−1 (−3, 0)^*^
*P*-value^#^ (post-pre)	**< 0.01**		**0.02**		**0.21**		**< 0.01**		**< 0.01**		**0.04**	

* p < 0.05; The P-value was calculated using the Wilcoxon signed-rank test to compare pre- and post-therapy.

# The P-value was calculated using the Wilcoxon test to compare the two groups.

Data are presented as median (P25, P75).

OABSS, overactive bladder symptom score; QoL score, quality of life score.

### 3.3. Adverse events

All 46 patients completed either micro-RF or oral tolterodine treatment, as well as the complete follow-up. The incidence of adverse events in the micro-RF group was 8.7% (2/23) and that in the control group was 43.5% (10/23). The following two adverse events happened in the micro-RF group: an injury to the urethra during catheterization in a man and a urinary tract infection in a woman, both of which were relieved or disappeared after day 3. The adverse effects in the tolterodine group were mainly dry mouth (4/23), dysuria (5/23), and constipation (8/23), but none of the patients withdrew from the drug therapy.

## 4. Discussion

To the best of our knowledge, this is the first retrospective cohort study to compare the safety and efficacy between micro-RF therapy and tolterodine treatment for newly diagnosed moderate or severe OAB. Through a short-term follow-up, the micro-RF therapy showed better therapeutic efficacy and a lower and tolerable adverse event rate than oral tolterodine for newly diagnosed moderate-to-severe OAB.

Moderate-to-severe OAB has a significant impact on both patient quality of life and workforce productivity. Less invasive procedures are recommended as the first-line treatment for OAB therapy by the Consensus Conference on Urinary Incontinence in Adults and by the guidelines of the American Urological Association/Society of Urodynamics, Female Pelvic Medicine, and Urogenital Reconstruction (AUA/SUFU) (Gormley et al., 2015; Marti et al., 2015). Nevertheless, antimuscarinic agents are usually the first option of therapies considered by urologists for treating OAB in clinical practice (Marti et al., 2015), but a large proportion of patients stop taking these drugs due to poor compliance or drug intolerance. In this study, patients who accepted micro-RF therapy had a success rate of 73.9% at the 7th week of follow-up, which was significantly higher than 43.5% of tolterodine treatment. For the secondary efficacy indicators, daily voiding times, daily urgency episodes, daily UI episodes, mean volume per micturition, OABSS, and QoL score all markedly improved after micro-RF therapy, and the magnitude of improvement was higher than in the tolterodine group. Moreover, no unacceptable complications were observed in the micro-RF therapy group. The incidence of adverse reactions related to micro-RF therapy was 8.7%, which was much less than the 43.5% of tolterodine treatment. The significant efficacy and non/mini-invasive characteristics make micro-RF therapy a potential first-line treatment option for newly diagnosed OAB, which needs to be verified through a strictly designed, prospective clinical trial.

It was reported that a modified Ingelman-Sundberg procedure that blocks the terminal pelvic nerve branches to the bladder showed satisfactory efficacy for women with urge incontinence and intractable detrusor instability (Cespedes et al., 1996). To achieve a similar therapeutic effect while avoiding the inherent risks of surgery, Fugett et al. (2018) recently proved that a selective bladder denervation device could provide a targeted trigone ablation with resultant denervation, and the preliminary safety was confirmed in extirpated ovine bladder trigones. It also has been confirmed that modulated radiofrequency ablation delivered by their novel ablation device can reduce nerve density in the bladder neck and trigone by 88.6 and 88.9% at 12 weeks without evidence of lasting epithelial injury (Okhunov et al., 2019). These studies suggest bladder radiofrequency therapy is a feasible therapeutic option to modify and disrupt nerve signaling pathways in the human bladder.

Traditional high-temperature RF, as a minimally invasive therapy method, has been widely applied in treating multiple diseases, such as atrial fibrillation (Parameswaran et al., 2021), chronic vertebrogenic low back pain (Ren et al., 2021), and primary trigeminal neuralgia (Urits et al., 2021). Rovner et al. (2019) recently found that a single treatment with selective bladder denervation is durable for 1 year in a significant proportion of women with refractory OAB. However, the micro-RF therapy performed in this study is quite different from traditional radiofrequency ablation. First, the precise energy is transferred onto the mucosa of the trigone of the bladder through an electrode slice enclosing a distal catheter, which is a novel delivery antenna system. Besides, the micro-RF power is controlled under 10 W, maintaining the temperature below 45°C, which is significantly lower than the 60°C of traditional RF (Xu et al., 2022) and thus would not cause bladder mucosa burns and related complications.

There are still several limitations to our study. First, as a single-center retrospective study, the reliability of the conclusions needs to be further verified through a well-designed prospective and randomized controlled study. Second, the small cohort size may affect the statistical power to detect significant differences between groups. Moreover, the results of this study were based on a 7-week follow-up, and a long-term observational study is required to confirm our conclusion.

## 5. Conclusion

Through this retrospective study, we found that micro-RF therapy is safer and more effective than oral tolterodine for newly diagnosed moderate-to-severe OAB in a short-term follow-up. In our center, a prospective and randomized controlled trial is being conducted to evaluate whether micro-RF therapy could replace pharmacological therapy and be applied to newly diagnosed patients with moderate-to-severe OAB as first-line treatment.

## Data availability statement

The raw data supporting the conclusions of this article will be made available by the authors, without undue reservation.

## Ethics statement

The studies involving human participants were reviewed and approved by the Ethics Committee of the First Affiliated Hospital of Nanjing Medical University (No. 2022-SR-569) . The patients/participants provided their written informed consent to participate in this study.

## Author contributions

XM: conceptualization, methodology, and funding acquisition. MT: data curation, writing the original draft preparation, and funding acquisition. PL: investigation, formal analysis, writing, reviewing, and editing. CW: data analysis and interpretation. QZ: collection and assembly of data. All authors did the final approval of manuscript.
